# Combined elevation of pre-treatment γ-glutamyltransferase and lactate dehydrogenase as independent prognosticator for metastatic renal cell carcinoma undergoing immune-based therapy

**DOI:** 10.1038/s41598-026-48270-3

**Published:** 2026-04-18

**Authors:** Annemarie Uhlig, Angelika Mattigk, Marcus Sondermann, Benedikt Hoeh, Cristina Cano Garcia, Niklas Klümper, Alexander Cox, Oliver Hahn, Philipp Schmucker, Luka Flegar, Jonathan Vollemaere, Friedemann Zengerling, Severine Banek, Jörg Ellinger, Johannes Huber, Philip Zeuschner, Charis Kalogirou, Kati Erdmann

**Affiliations:** 1 Department of Urology, University Hospital Tübingen, Tübingen, Germany; 2 Department of Urology and Paediatric Urology, University Hospital Ulm, Ulm, Germany; 3 Department of Urology, University Hospital Carl Gustav Carus, Technische Universität Dresden, Dresden, Germany; 4 Department of Urology, University Hospital Frankfurt, Goethe University Frankfurt am Main, Frankfurt, Germany; 5 Institute of Experimental Oncology, University Hospital Bonn, Bonn, Germany; 6 Department of Urology, University Hospital Bonn, Bonn, Germany; 7 Department of Urology and Paediatric Urology, Julius Maximilians University Medical Center of Würzburg, Würzburg, Germany; 8 Department of Urology, University Hospital Freiburg, Freiburg, Germany; 9 Department of Urology, Philipps-University Marburg, Marburg, Germany; 10 Department of Urology and Paediatric Urology, Saarland University, Homburg/Saar, Germany

**Keywords:** γ-glutamyltransferase, Immune checkpoint inhibitors, Lactate dehydrogenase, Prognostic biomarkers, Metastatic renal cell cancer, Prognostic markers, Translational research, Renal cell carcinoma, Renal cell carcinoma, Cancer metabolism, Cancer immunotherapy

## Abstract

In a preceding monocentric study, serum liver enzymes (ALAT, ASAT, GGT) and lactate dehydrogenase (LDH) were identified as independent prognosticators for patients with metastatic renal cell cancer (mRCC) treated with immune-based first-line (1L) therapy. Here, we evaluated the prognostic potential of ALAT, ASAT, GGT and LDH in a larger multicentric real-world cohort. Baseline levels of ALAT, ASAT, GGT and LDH from 240 patients with mRCC undergoing immune-based 1L therapy were retrospectively investigated regarding progression-free survival (PFS), overall survival (OS) and response rates. Independent prognostic factors for PFS and OS were identified via multivariate Cox regression analyses. Although none of the serum parameters were markedly associated with therapy response, patients with low LDH showed a significantly longer PFS. Furthermore, patients with low GGT and LDH also exhibited a significantly prolonged OS. The combination of GGT and LDH risk groups enhanced the prognostic power. The concomitant elevation of both GGT and LDH was significantly associated with a higher progression rate and increased overall mortality and subsequently identified as independent prognosticator for PFS and OS. Overall, the prognostic potential of baseline GGT and LDH serum levels was substantiated in a larger multicentric cohort of patients with mRCC undergoing immune-based 1L therapy.

**Supplementary Information:**

The online version contains supplementary material available at 10.1038/s41598-026-48270-3.

## Introduction

Renal cell carcinoma (RCC) accounts for 2% of global cancer diagnoses and deaths with an annual incidence of about 435,000 cases worldwide and 156,000 cancer deaths per year^1^. Over the past decade, immune-based therapy with checkpoint inhibitors (CPI) has become a cornerstone in the treatment of metastatic RCC (mRCC)^2,3^. First-line (1L) therapy of treatment-naïve mRCC consists of either a combination of two CPIs or a combination of a CPI with a tyrosine kinase inhibitor (TKI)^3,4^. In the updated guideline of the European Association of Urology, CPI-based combination therapies can be offered to patients with mRCC irrespective of their International mRCC Database Consortium (IMDC) risk group^4^. TKI monotherapy, e.g., with sunitinib or pazopanib, is indicated in patients with favorable IMDC risk or when CPI-based therapy is not an option^4^. After prior TKI monotherapy, CPI monotherapy with nivolumab is recommended in subsequent treatment lines (≥ 2L)^4^. Although CPI-based 1L therapy demonstrates improved survival outcomes, treatment response remains highly variable^5^, underscoring the need for reliable prognostic biomarkers for progression-free survival (PFS) and overall survival (OS) in affected patients.

Liver function parameters like alanine aminotransferase (ALAT), aspartate aminotransferase (ASAT) and γ-glutamyltransferase (GGT) as well as the tissue damage marker lactate dehydrogenase (LDH) reflect tumor burden, systemic inflammation and metabolic activity and thereby emerged as potential prognostic indicators in various malignancies^6–11^. Understanding the impact of these biomarkers on survival outcomes in patients with mRCC undergoing CPI-based therapy may provide valuable insights for risk stratification and treatment optimization. In a preceding monocentric study comprising 82 patients, ALAT, ASAT, GGT and LDH were identified as independent prognostic factors for patients with mRCC treated with CPI-based 1L therapy^12^. High baseline levels of ALAT, ASAT, GGT and LDH were significantly associated with shorter PFS and OS, whereas the De Ritis-Ratio (DRR = ASAT/ALAT) exhibited no prognostic value. Following the combination of the individual markers, patients with simultaneously high levels of ALAT, ASAT, GGT and LDH displayed lower response rates and poor survival with the highest risk for progression or death^12^. Consequently, liver enzymes and LDH may serve as accessible tools for therapy monitoring of patients with mRCC undergoing CPI-based 1L therapy since they are readily available in the clinical routine.

In this study, we evaluated the prognostic potential of liver enzymes and LDH in a larger multicentric real-world cohort from five university hospitals in Germany. Baseline levels of ALAT, ASAT, GGT and LDH from 240 patients with mRCC treated with CPI-based 1L therapy were retrospectively investigated regarding their association with treatment outcome including survival and response rates.

## Materials and methods

### Patients

We retrospectively included 258 patients with mRCC from five university hospitals in Germany who underwent CPI-based combination therapy between 2017 and 2024. Patients’ demographics as well as clinical and laboratory parameters were retrospectively retrieved from their medical records (censor date 31 July 2024). Patients with a follow up < 1 month (*n* = 6), with missing (*n* = 4) or insufficient (*n* = 6) values for the assessed laboratory parameters and without CPI treatment (*n* = 2) were excluded from further analysis resulting in a final study cohort of 240 patients (Supplementary Fig. S1). Response to treatment was determined based on radiological assessment and classified as complete response (CR), partial response (PR), stable disease (SD) or progressive disease (PD). This study was approved by the Ethical Review Board of the Julius Maximilians University Medical Center of Würzburg (reference #20201211-01) and conducted according to the Declaration of Helsinki.

## Assessment of laboratory parameters

The serum levels of ALAT, ASAT, GGT and LDH were routinely determined during patients’ appointments at the respective study center. Measurements were obtained from certified clinical chemistry laboratories, each adhering to internal and external quality assurance programs according to national standards. Although minor inter-laboratory variations in assay methods and reference ranges may exist, all values were expressed in or converted to uniform international units (U/l). The baseline values for each parameter including the calculated DRR (ASAT/ALAT ratio) were sampled within 41 days prior to and up to seven days after treatment initiation (median 0 days), whereupon the majority of the baseline data (*n* = 208, 86.7%) were measured within the interval of -7 to + 7 days. For survival analyses, all parameters were then dichotomized at the median and classified as “high” vs. “low” as previously described^12,13^. Ultimately, categorized GGT and LDH baseline levels were combined with the following groups: zero risk factors = both GGT and LDH low, one risk factor = either GGT or LDH high, two risk factors = both GGT and LDH high.

### Statistical analysis

IBM SPSS Statistics 30.0.0.0 (IBM, Armonk, NY, USA) and GraphPad Prism 10.2.3 (GraphPad Software, San Diego, CA, USA) were used for statistical analyses. Differences of continuous parameters between two or three groups were evaluated by the Mann-Whitney-U test or Kruskal-Wallis test, respectively. The strength of the linear relationship between two laboratory parameters was examined by determination of the Pearson correlation coefficient (r). PFS was defined as time from treatment start until radiological or clinical disease progression or death from any cause while still on 1L therapy. OS was defined as the time from 1L start to date of death from any cause. Patients without an event were censored at the date with the last confirmed survival. The Kaplan-Meier method with the log-rank test and life tables were used to delineate survival differences between the different risk groups and to calculate survival proportions, respectively. Hazard ratios (HR) including their 95% confidence intervals (95%CI) as well as Harrell’s concordance indices (C-index) were determined by univariate or multivariate Cox regression analyses. The latter was conducted by including parameters with *p* values < 0.05 in univariate analyses in order to identify independent prognostic factors. Statistical significance was set at *p* < 0.05.

## Results

### Patient cohort

A total of 240 patients (median patient age 68 years, 28% female) were included (Table 1). At 1L start, most patients had an Eastern Cooperative Oncology Group performance status (ECOG) of ≥ 1 (*n* = 139, 58%) and an intermediate IMDC risk (*n* = 139, 58%). CPI-based 1L therapy was initiated at a median of 9.5 months after first RCC diagnosis. The predominant histology was clear cell RCC (*n* = 192, 80%) followed by the papillary subtype (*n* = 16, 7%). Most patients (*n* = 169, 70%) had received a partial or radical nephrectomy in the past. A total of 73 patients (30%) had single organ metastases, the remainder multiple metastases (70%). Liver metastases were present in 42 patients (18%). Type of treatment was CPI + CPI for 73 patients (30%) and CPI + TKI for 167 patients (70%) with pembrolizumab + axitinib (34%) being the most common combination.

**Table 1:** Characteristics of 240 patients with mRCC undergoing CPI-based 1L therapy.

Parameter	Category	Total cohort (*n* = 240)
Age at 1L (years)	Median (range)	68 (19–88)
Sex (n)	Male Female	173 67
ECOG at 1L (n)	0 1 2 3 Unknown	93 105 28 6 8
IMDC risk at 1L (n)	Favorable Intermediate Poor Unknown	44 139 56 1
Time from RCC diagnosis to 1L (months)	Median (range)	9.5 (0-341)
T stage at RCC diagnosis (n)^a^	T1-2 T3-4 Unknown	81 116 43
N stage at RCC diagnosis (n)^a^	N0 N1 Unknown	85 55 100
M stage at RCC diagnosis (n)^a^	M0 M1 Unknown	88 124 28
Histology grade at RCC diagnosis (n)	G1-2 G3-4 Unknown	82 96 62
Histology at RCC diagnosis (n)	Clear cell Papillary Chromophobe Sarcomatoid Clear cell mixed Others or unknown	192 16 3 3 9 15
Nephrectomy prior 1L (n)	No Yes	71 169
Metastatic organs at 1L (n)	Single Multiple	73 167
Liver metastasis at 1L (n)	No Yes	198 42
1L therapy (n)	Avelumab+axitinib Nivolumab+cabozantinib Nivolumab+ipilimumab Pembrolizumab+axitinib Pembrolizumab+lenvatinib	19 27 73 82 39
CPI cycles	Median (range)	10 (1–91)
Follow-up duration (months)	Median (range)	18 (1–62)
BOR during 1L (n)	CR PR SD PD Unknown	9 99 71 45 16

^a^Combined clinical and pathological stage.

The median follow-up was 18 months. Overall, 149 patients progressed or died during 1L therapy, resulting in a median PFS of 13 months (95%CI 10.2–15.8 months). Treatment response was available for 224 patients with the following best overall responses (BOR): CR *n* = 9 (4%), PR *n* = 99 (44%), SD *n* = 71 (32%) and PD *n* = 45 (20%). At the censor date (July 2024), 95 patients had died and the median OS of the total cohort was 38 months (95%CI 30.1–45.9 months).

### Baseline levels of ALAT, ASAT, DRR, LDH and GGT

The median baseline levels were 19 U/l (= 0.32 µmol/s*l) for ALAT, 22 U/l (= 0.37 µmol/s*l) for ASAT, 1.18 for DRR, 34.2 U/l (= 0.58 µmol/s*l) for GGT and 222 U/l (= 3.7 µmol/s*l) for LDH (Table 2). The median baseline levels of all parameters did not differ significantly between study centers (Kruskal-Wallis test: *p* > 0.05; data now shown). Except for a high correlation between baseline ALAT and ASAT levels (*r* = 0.691, *p* < 0.001), the other parameters correlated only weakly (*r* < 0.5; Supplementary Table S1). None of the parameters was significantly associated with the presence of liver metastasis (Supplementary Table S2).

**Table 2:** Median baseline levels of ALAT, ASAT, DRR, GGT and LDH in the overall cohort and depending on the response groups.

Parameter	Category	Overall	CR	PR	SD	PD	*p* value ^a^
ALAT (U/l)	n	237	9	96	71	45	
	Median (range)	19.0 (5.0-115.8)	18.0 (9.6–58.8)	18.0 (5.4–94.0)	20.0 (5.0-115.8)	21.0 (7.8–113.0)	0.694
ASAT (U/l)	n	206	7	84	64	37	
	Median (range)	22.0 (8.4-163.2)	22.8 (12.6–36.0)	21.8 (8.4-163.2)	23.2 (11.4-145.2)	21.0 (13.0-159.0)	0.625
DRR	n	203	7	81	64	37	
	Median (range)	1.18 (0.27–4.40)	1.28 (0.56–1.62)	1.18 (0.48–3.44)	1.09 (0.27-4.00)	1.19 (0.44–2.40)	0.633
GGT (U/l)	n	239	9	99	70	45	
	Median (range)	34.2 (10.0-682.0)	25.2 (11.4–167.0)	34.8 (11.4–637.0)	34.1 (13.8-567.6)	37.8 (16.0-682.0)	0.316
LDH (U/l)	n	234	9	96	70	44	
	Median (range)	222 (44–989)	197 (158–389)	223 (132–576)	223 (44–369)	222 (118–989)	0.414

^a^Comparison between response groups by the Kruskal-Wallis test (CR vs. PR vs. SD vs. PD).

## Progression-free survival and response

**Fig. 1:**
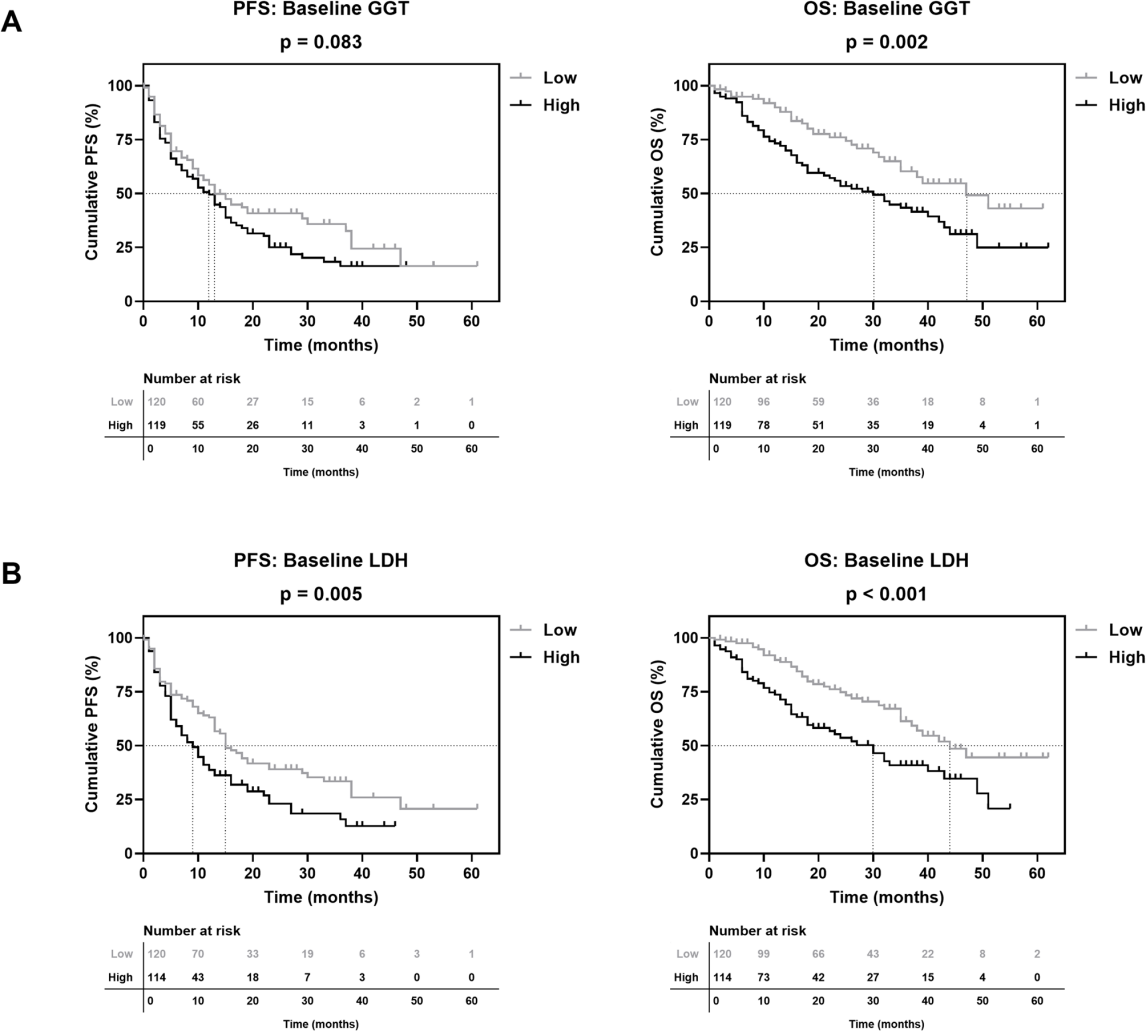
Association of baseline (**A**) GGT and (**B**) LDH with PFS and OS of patients with mRCC undergoing CPI-based 1L therapy. Vertical dashed lines indicate the respective median survival time of each category. *P* values were calculated using the log-rank test.

Patients with low LDH levels showed a significantly longer PFS than the group with high LDH (15 vs. 9 months), resulting in higher 1-year and 2-year survival rates (64% vs. 41% & 39% vs. 23%; Fig. 1 & Supplementary Table S3). Furthermore, patients with low GGT (13 vs. 12 months) and low ALAT (15 vs. 10 months) tended to have a longer PFS, although this did not reach statistical significance (Fig. 1, Supplementary Fig. S2, Supplementary Table S3). No statistically significant difference in PFS was also found for high versus low ASAT and DRR (Supplementary Fig. S2, Supplementary Table S3). None of the parameters exhibited a significant association with response groups (Table 2).

Via univariate Cox regression analysis, high LDH (HR = 1.58, 95%CI 1.13–2.19) was identified as potential risk factor for progression or death during 1L therapy (Table 3). In addition, nephrectomy status, presence of liver metastasis and type of 1L therapy showed prognostic potential (Table 3, Supplementary Fig. S3). After co-adjusting all parameters with *p* < 0.05 in univariate analysis, high LDH emerged as independent prognostic factor via multivariate Cox regression analysis (HR = 1.51, 95%CI 1.08–2.11; Table 3). In addition, this multivariate model (nephrectomy status, presence of liver metastasis, type of 1L therapy, baseline LDH) exhibited a higher C-index (0.619) than the individual parameters (C-indices 0.542–0.564; Supplementary Table S4).

**Table 3:** Uni- and multivariate Cox regression analyses for PFS depending on clinico-pathological and baseline laboratory parameters.

Parameter	Category	Univariate		Multivariate	
		HR (95%CI)	*p* value	HR (95%CI)	*p* value
Age	Continuous	0.99 (0.98–1.01)	0.288		
Sex	Male Female	Reference 1.31 (0.93–1.84)	0.130		
ECOG	0 1–3	Reference 1.14 (0.81–1.59)	0.455		
IMDC risk	Favorable Intermediate Poor	Reference 0.67 (0.44–1.02) 1.02 (0.64–1.62)	0.060 0.945		
Prior nephrectomy	No Yes	Reference 0.69 (0.50–0.97)	**0.034**	Reference 0.77 (0.54–1.09)	0.135
Metastatic organs at 1L	Single Multiple	Reference 1.21 (0.84–1.74)	0.305		
Liver metastasis at 1L	No Yes	Reference 1.68 (1.14–2.48)	**0.009**	Reference 1.56 (1.06–2.32)	**0.026**
1L therapy	CPI + CPI CPI + TKI	Reference 0.69 (0.49–0.96)	**0.029**	Reference 0.67 (0.47–0.94)	**0.021**
Baseline ALAT	Low High	Reference 1.34 (0.97–1.85)	0.081		
Baseline ASAT	Low High	Reference 1.21 (0.85–1.73)	0.285		
Baseline DRR	Low High	Reference 0.91 (0.64–1.30)	0.600		
Baseline GGT	Low High	Reference 1.32 (0.95–1.83)	0.093		
Baseline LDH	Low High	Reference 1.58 (1.13–2.19)	**0.007**	Reference 1.51 (1.08–2.11)	**0.017**

Significant *p* values (< 0.05) are displayed in bold. Only parameters with *p* < 0.05 in the univariate analysis were included in multivariate Cox regression analyses. CI: confidence interval, HR: hazard ratio.

## Overall survival

Patients with low GGT and LDH also exhibited a significantly prolonged OS compared to the respective group with high baseline levels (GGT: 47 vs. 30 months, LDH: 44 vs. 30 months; Fig. 1). This was associated with higher 1-year and 2-year survival rates (GGT: 92% vs. 74% & 75% vs. 56%, LDH: 92% vs. 74% & 76% vs. 55%; Supplementary Table S3). No statistically significant difference in OS was found for high versus low ASAT, ALAT and DRR (Supplementary Fig. S2, Supplementary Table S3).

Univariate Cox regression analysis identified high GGT (HR = 1.92, 95%CI 1.26–2.92) and high LDH (HR = 1.99, 95%CI 1.31–3.02) as risk factors for death (Table 4). Furthermore, IMDC risk group, nephrectomy status and presence of liver metastasis displayed prognostic potential (Table 4, Supplementary Fig. S3). Subsequently, high GGT (HR = 1.77, 95%CI 1.14–2.73) and high LDH (HR = 1.77, 95%CI 1.15–2.73) emerged as independent prognosticators for OS in multivariate Cox regression analyses including all parameters with *p* < 0.05 in univariate analysis (Table 4). With a C-index of 0.693, this multivariate model (IMDC risk group, nephrectomy status, presence of liver metastasis, baseline GGT, baseline LDH) also showed a higher prognostic power than the individual parameters (C-indices 0.566–0.610; Supplementary Table S4).

**Table 4:** Uni- and multivariate Cox regression analyses for OS depending on clinico-pathological and baseline laboratory parameters.

Parameter	Category	univariate		multivariate	
		HR (95%CI)	*p* value	HR (95%CI)	*p* value
Age	Continuous	1.00 (0.98–1.03)	0.697		
Sex	Male Female	Reference 1.31 (0.85–2.01)	0.229		
ECOG	0 1–3	Reference 1.31 (0.86–1.99)	0.215		
IMDC risk	Favorable Intermediate Poor	Reference 1.84 (0.98–3.46) 3.21 (1.63–6.31)	0.059 **< 0.001**	Reference 1.65 (0.87–3.12) 2.30 (1.13–4.69)	0.122 **0.022**
Prior nephrectomy	No Yes	Reference 0.48 (0.32–0.73)	**< 0.001**	Reference 0.61 (0.39–0.97)	**0.036**
Metastatic organs at 1L	Single Multiple	Reference 1.35 (0.85–2.13)	0.203		
Liver metastasis at 1L	No Yes	Reference 2.00 (1.24–3.22)	**0.005**	Reference 1.74 (1.06–2.85)	**0.029**
1L therapy	CPI + CPI CPI + TKI	Reference 0.81 (0.54–1.23)	0.325		
Baseline ALAT	Low High	Reference 1.13 (0.75–1.70)	0.564		
Baseline ASAT	Low High	Reference 1.25 (0.81–1.94)	0.318		
Baseline DRR	Low High	Reference 1.30 (0.83–2.04)	0.250		
Baseline GGT	Low High	Reference 1.92 (1.26–2.92)	**0.002**	Reference 1.77 (1.14–2.73)	**0.010**
Baseline LDH	Low High	Reference 1.99 (1.31–3.02)	**0.001**	Reference 1.77 (1.15–2.73)	**0.010**

Significant *p* values (< 0.05) are displayed in bold. Only parameters with *p* < 0.05 in the univariate analysis were included in multivariate Cox regression analyses. CI: confidence interval, HR: hazard ratio.

### Prognostic value of combined GGT and LDH risk groups

**Fig. 2:**
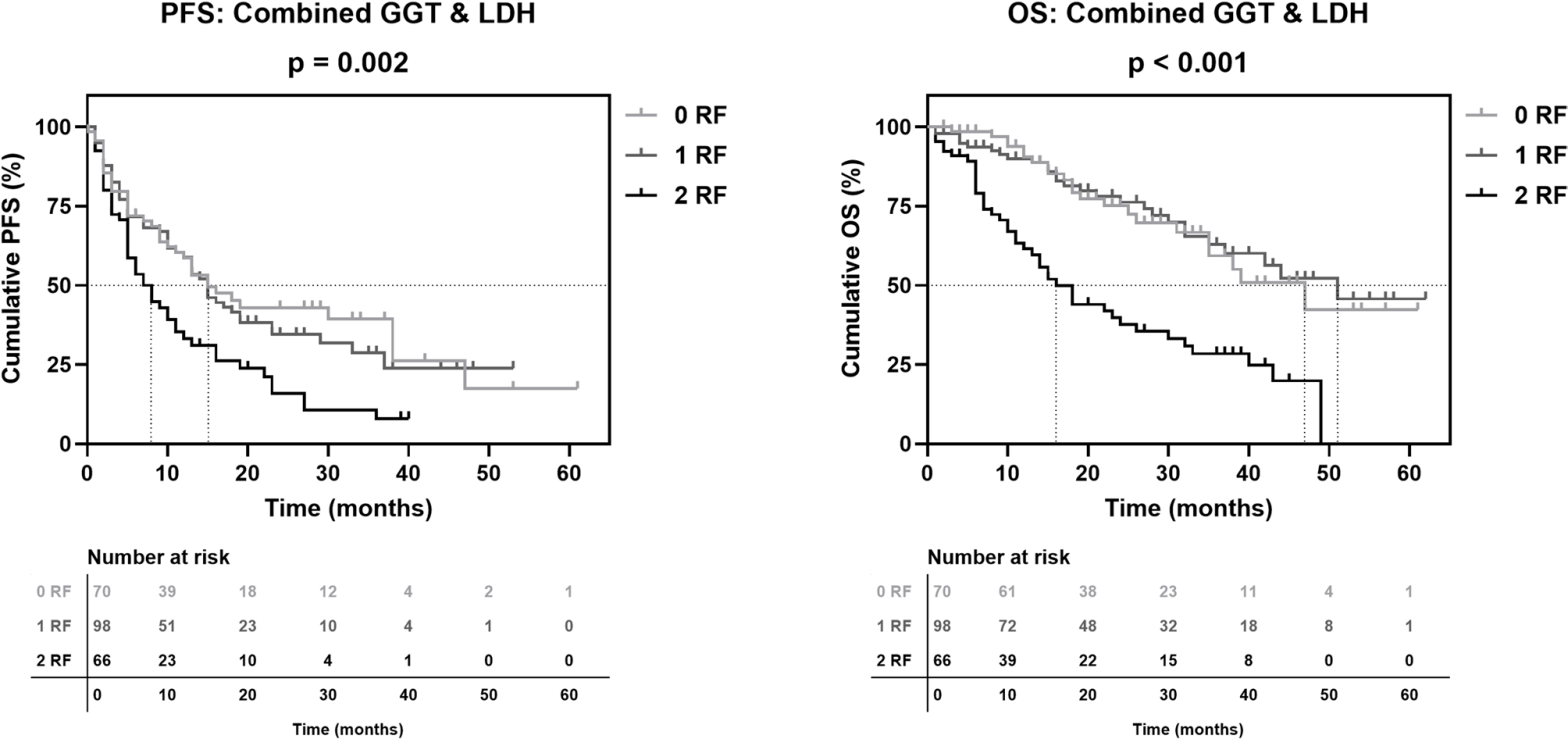
Association of the combined baseline GGT/LDH risk groups with PFS and OS of patients with mRCC undergoing CPI-based 1L therapy. A risk factor (RF) is defined as either high GGT or LDH. Vertical dashed lines indicate the respective median survival time of each category. *P* values were calculated using the log-rank test.

Since baseline GGT and LDH were identified as independent prognosticators for PFS and/or OS, patients were further stratified according to their combined baseline GGT/LDH risk groups: zero risk factors = both GGT and LDH low, one risk factor = either GGT or LDH high, two risk factors = both GGT and LDH high. Patients with both risk factors present showed significantly worse PFS and OS than patients with only one or none risk factor (PFS: 8 vs. 15 vs. 15 months, OS: 16 vs. 51 vs. 47 months; Fig. 2 & Supplementary Table S3). This association with survival was reflected by lower 1-year and 2-year survival rates for PFS (35% vs. 60% vs. 60% & 16% vs. 34% vs. 42%) and OS (63% vs. 90% vs. 94% & 40% vs. 78% vs. 75%; Supplementary Table S3). Patients with combined elevation of GGT and LDH also exhibited significantly worse PFS and OS in the CPI + TKI subgroup, whereas no association with survival was observed in the CPI + CPI subgroup (Supplementary Fig. S4). Furthermore, patients in the subgroup with poor IMDC risk had a significantly worse PFS when GGT and LDH were increased (Supplementary Fig. S5). In contrast, simultaneous elevation of GGT and LDH was associated with worse OS regardless of IMDC risk group, although statistical significance was only reached in the subgroups with good or poor IMDC risk (Supplementary Fig. S5).

In univariate Cox regression analyses, the presence of two risk factors (high GGT and LDH) was also significantly associated with a higher progression rate (HR = 1.92, 95%CI = 1.26–2.93, *p* = 0.002) and increased overall mortality (HR = 2.96, 95%CI = 1.76–4.97, *p* < 0.001). Subsequently, the combined elevation of GGT and LDH was identified as independent prognostic factor for PFS (HR = 1.84, 95%CI 1.19–2.84) and OS (HR = 2.82, 95%CI 1.67–4.77) in multivariate Cox regression analyses (Table 5). With C-indices of 0.564 for PFS and 0.642 for OS, the prognostic value of the combined GGT/LDH score was similar to or even higher than those of the clinico-pathological parameters (C-indices for PFS 0.542–0.564, C-indices for OS 0.566–0.610; Supplementary Table S4). When the clinico-pathological parameters were co-adjusted with the combined GGT/LDH score, the prognostic ability was even further enhanced resulting in C-indices of 0.633 for PFS and 0.704 for OS (Supplementary Table S4).

**Table 5:** Multivariate Cox regression analyses for PFS and OS depending on selected clinico-pathological and the combined baseline GGT/LDH risk groups.

Parameter	Category	PFS		OS	
		HR (95%CI)	*p* value	HR (95%CI)	*p* value
IMDC risk	Favorable Intermediate Poor			Reference 1.57 (0.83–2.97) 2.06 (1.00-4.23)	0.163 **0.049**
Prior nephrectomy	No Yes	Reference 0.77 (0.55–1.10)	0.150	Reference 0.61 (0.39–0.96)	**0.033**
Liver metastasis at 1L	No Yes	Reference 1.51 (1.01–2.26)	**0.046**	Reference 1.65 (1.00-2.71)	**0.049**
1L therapy	CPI + CPI CPI + TKI	Reference 0.59 (0.41–0.86)	**0.022**		
Combined GGT & LDH	0 RF 1 RF 2 RF	Reference 1.24 (0.81–1.89) 1.84 (1.19–2.84)	0.322 **0.006**	Reference 1.07 (0.61–1.90) 2.82 (1.67–4.77)	0.807 **< 0.001**

A risk factor (RF) is defined as either high GGT or LDH. Significant *p* values (< 0.05) are displayed in bold. Only clinico-pathological parameters with *p* < 0.05 in univariate analyses (Tables 3 & 4) were included in multivariate Cox regression analyses. CI: confidence interval, HR: hazard ratio, RF: risk factor.

## Discussion

This multicentric, retrospective study analyzed 240 patients with mRCC treated with CPI-based 1L therapy to evaluate the prognostic value of serum liver enzymes and LDH. High baseline levels of LDH and GGT were independently associated with shorter PFS and/or OS. Stratification by combined GGT/LDH risk groups further enhanced prognostic discrimination: patients with both markers elevated had significantly worse survival outcomes.

### Treatment and response patterns

Patients in the study were treated with a range of CPI-based combination therapies, which resulted in varied outcomes with 4% CR, 44% PR and 32% SD. Only 20% experienced PD reflecting the efficacy of CPI-based therapies in mRCC. However, the objective response rate (CR + PR: 48%) was slightly worse than in the setting of clinical trials^14–17^, which can be expected in a real-world setting.

Baseline values of ALAT, ASAT, DRR, GGT and LDH were also examined in relation to treatment response. However, no significant differences in baseline levels of these markers were observed across the response groups. This is in contrast to the preceding study, where response to therapy (CR + PR) was more frequent in patients with low baseline levels of ALAT, ASAT, GGT and LDH^12^. Overall, this suggests that while these biomarkers are often implicated in liver function and systemic inflammation, they may not serve as reliable standalone biomarkers for response in this cohort, underlining the complexity of immune-related response mechanisms^18,19^.

### Clinical parameters as prognostic factors for PFS and OS

Among the clinical parameters, IMDC risk category, nephrectomy status, presence of liver metastasis and type of combination therapy emerged as prognostic factors for PFS and/or OS, which aligns with findings from other real-world cohorts^20–22^. Patients with past nephrectomy exhibited improved survival, while poor IMDC risk was linked to increased overall mortality. Liver metastasis was significantly associated with reduced survival, corroborating its role as a negative prognostic factor in mRCC^20,23,24^. The presence of liver involvement often correlates with a more aggressive disease course and poorer response to CPI-based therapy^20,24^. Furthermore, the results also indicated that CPI + TKI combinations provided superior PFS compared to dual CPI therapies. Advantageous outcomes for treatments with CPI + TKI have also been reported in other real-world cohorts of mRCC^20–22^.

### Baseline LDH and GGT as prognostic factors for PFS and OS

In the preceding monocentric study, high baseline levels of ALAT, ASAT, GGT and LDH were independently associated with a higher progression rate and increased overall mortality in patients with mRCC treated with CPI-based 1L therapy^12^. Particularly for OS, GGT and LDH displayed a higher prognostic value than the investigated clinical prognostic factors. Here, we could corroborate GGT and LDH as independent prognosticators in a larger, multicentric cohort. High LDH levels at baseline were significantly associated with worse PFS and OS outcomes, suggesting that LDH might be a reliable marker of tumor burden and systemic inflammation in patients with mRCC treated with CPI-based 1L therapies. Similarly, elevated GGT levels correlated with poor OS, further emphasizing the association of liver function markers with oncological outcomes in patients with mRCC undergoing CPI-based 1L therapy. In contrast, the prognostic value of ALAT and ASAT could not be confirmed in the present study. In accordance with the preceding study^12^, the DRR as the ASAT/ALAT ratio was without prognostic significance.

In accordance with our findings, high levels of GGT and LDH were also associated with worse survival in patients with mRCC receiving ≥2L nivolumab^13,25,26^ as well as in CPI-treated melanoma, lung cancer and hepatocellular cancer^27–30^. GGT and LDH are involved in cancer metabolism and thus might reflect tumor burden and systemic inflammation^6–8^. Via conversion of pyruvate to lactate, LDH restores NAD^+^ for ongoing aerobic glycolysis (Warburg effect), which provides sufficient energy and key nutrients for cancer cell proliferation^7^. GGT is involved in the metabolism of the antioxidant glutathione and can promote carcinogenesis and tumor progression^6^. Both GGT and LDH have also been shown to exert immunosuppressive effects^8,31^ and thus could influence efficacy of immunotherapy.

Notably, the combination of GGT and LDH risk groups enhanced their prognostic power. The presence of both high GGT and LDH levels was significantly and independently associated with a higher progression rate and increased overall mortality. Particularly for OS, the combined GGT/LDH score outperformed the prognostic value of clinico-pathological parameters, such as IMDC risk, and added additional prognostic value in multivariate models as indicated by higher C-indices. In accordance, prognostic models including GGT and LDH better stratified patients with advanced pancreatic ductal adenocarcinoma treated with chemotherapy^32^ and urothelial carcinoma of the upper urinary tract after radical nephroureterectomy^33^ regarding their prognosis. The combined elevation of GGT and LDH might reflect increased oxidative stress and a changed energy metabolism due to their role in glutathione metabolism and glycolysis, respectively^6–8^.

In subgroup analysis regarding therapy regimen, a significant association of the combined GGT/LDH score with prognosis could only be observed in the CPI + TKI treatment group, but not in the CPI + CPI subgroup. These observations may be explained by a combination of biological and statistical factors: The subgroup of patients treated with CPI + CPI was smaller than the CPI + TKI subgroup (72 vs. 162). Thus, statistical testing might be underpowered. On the other hand, we detailed above that LDH and GGT reflect metabolic aggressiveness and oxidative stress, which are particularly relevant for the efficacy of TKI-based regimens that target hypoxia-driven angiogenesis and tumor metabolism. The prognostic impact of combined GGT/LDH therefore appears more pronounced in CPI + TKI-treated patients than in those receiving CPI + CPI.

Furthermore, the combined GGT/LDH score showed prognostic potential for PFS in the subgroup with poor IMDC risk, whereas a prognostic association with OS could be demonstrated regardless of IMDC risk, albeit reaching statistical significance only in the subgroups with good and poor IMDC risk. In the IMDC good- and poor-risk categories, the GGT/LDH score is likely capturing either unexpectedly aggressive biology (good risk) or amplifying already pronounced metabolic dysregulation (poor risk), resulting in larger effect sizes. In contrast, intermediate-risk patients represent a more heterogeneous biological group in which the discriminative power of GGT and LDH is reduced. Taken together, these biological considerations, combined with differences in sample size and effect magnitude across subgroups, likely explain the observed pattern.

From a clinical perspective, the combined GGT/LDH score may serve as a simple, inexpensive and routinely available biomarker-based tool to support risk stratification. Patients presenting with both elevated GGT and LDH at the beginning of therapy could be recognized as having a particularly unfavorable prognosis and may benefit from intensified monitoring such as early imaging re-evaluation. Thereby, non-responders undergoing progression could potentially be identified earlier, and thus, therapy adjustments could be initiated without delay. Conversely, patients with low baseline levels of both markers might represent a subgroup with more favorable outcomes and could potentially be managed with standard follow-up intervals. Therefore, the GGT/LDH score could help refine personalized treatment strategies and improve patient counseling in the real-world management of mRCC.

### Limitations

Owing to its retrospective design, our study is not devoid of limitations. There could be a selection bias due to the exclusion of patients with missing or insufficient values for the assessed laboratory parameters. However, only 18 (7%) patients were excluded from further analysis from the originally 258 screened patients (Supplementary Fig. S1). Furthermore, biomarker distributions did not differ significantly among study centers.

Another potential limitation could be inter-laboratory variability in GGT and LDH assays across participating centers. However, all laboratories were accredited and followed standardized quality control procedures, and no significant differences in baseline median values could be observed between study centers. Prognostic groupings for each parameter (high vs. low) were defined based on the overall cohort median, which guarantees comparable group sizes for statistical analyses and minimizes bias from site-specific differences. However, this approach may not reflect optimal clinical thresholds, and consequently, the optimal cutoff values for GGT and LDH need to be prospectively determined. Nevertheless, the median values of GGT (34.2 U/l) and LDH (222 U/l) concurred with those in the preceding smaller monocentric study^12^ and were similar to the cutoff values used in other studies investigating the prognostic impact of both parameters in CPI-treated cancer patients (GGT: 42–71 U/l, LDH: 196–264 U/l) ^13,25–30^.

Baseline values were sampled within the time frame of − 41 to + 7 days relative to treatment initiation, which could potentially introduce minor variability into the analyses as laboratory values may have changed during this interval. However, most values (*n* = 208, 86.7%) were obtained within the interval of -7 to + 7 days around treatment start, and thus, the influence of timing heterogeneity on the results is expected to be limited.

Furthermore, the absence of a centralized review of radiological imaging could introduce some variability in the determination of progression and response. Potential confounding factors (e.g., liver disease, alcohol use, concurrent medications), which could influence GGT and LDH levels, were not comprehensively recorded as well. Therefore, the findings should be interpreted with caution in the context of a retrospective multi-institutional study. Lastly, although the number of events per degree of freedom met commonly recommended thresholds for multivariable Cox regression, the OS model operated at the lower end of this range. Therefore, a minor risk of overfitting cannot be fully excluded.

Nevertheless, the present cohort included all available CPI-based 1L therapy options for mRCC and thus reflects the current therapeutic landscape for mRCC. Perspectively, baseline GGT and LDH levels could aid clinicians in risk stratification and therapeutic decision-making. A prospective validation in larger, independent cohorts is warranted to optimize the time frame for laboratory sampling, establish cutoff values and assess potential confounders, thereby confirming the utility of GGT and LDH as monitoring biomarkers in clinical practice.

## Conclusions

In this multicentric retrospective study, we could further substantiate the prognostic value of baseline serum levels of GGT and LDH for patients with mRCC undergoing CPI-based 1L therapy. Stratification based on combined GGT and LDH levels further enhanced prognostic discrimination, identifying a subset of patients with particularly poor outcomes, who should be monitored by a more stringent radiological staging schedule. These findings underscore the clinical potential of integrating routinely available laboratory parameters as accessible and cost-effective prognostic tools in the management of mRCC. Overall, the combination of such biomarkers with strong prognostic value offers a significant opportunity to enhance patient care and to enable a more personalized approach to cancer immunotherapy.

## Supplementary Information

Below is the link to the electronic supplementary material.

Supplementary Material 1

## Data Availability

The datasets generated during and/or analyzed during the current study are available from the corresponding author on reasonable request.

## References

[1] Bray, F. et al. Global cancer statistics 2022: GLOBOCAN estimates of incidence and mortality worldwide for 36 cancers in 185 countries. *Cancer J. Clin.* **74**, 229–263. 10.3322/caac.21834 (2024). 10.3322/caac.21834 38572751

[2] Xu, W. X., Atkins, M. B. & McDermott, D. F. Checkpoint inhibitor immunotherapy in kidney cancer. *Nat. Reviews Urol.* **17**, 137–150. 10.1038/s41585-020-0282-3 (2020). 10.1038/s41585-020-0282-3 32020040

[3] Bedke, J. et al. The 2021 updated European Association of Urology guidelines on renal cell carcinoma: Immune checkpoint inhibitor-based combination therapies for treatment-naive metastatic clear-cell renal cell carcinoma are standard of care. *Eur. Urol.* **80**, 393–397. 10.1016/j.eururo.2021.04.042 (2021). 34074559 10.1016/j.eururo.2021.04.042

[4] Bex, A. et al. European Association of Urology guidelines on renal cell carcinoma: The 2025 update. *Eur. Urol.*10.1016/j.eururo.2025.02.020 (2025). 40118739 10.1016/j.eururo.2025.02.020

[5] Sharma, P., Hu-Lieskovan, S., Wargo, J. A. & Ribas, A. Primary, adaptive, and acquired resistance to cancer immunotherapy. *Cell* **168**, 707–723. 10.1016/j.cell.2017.01.017 (2017). 28187290 10.1016/j.cell.2017.01.017 PMC5391692

[6] Takemura, K., Board, P. G. & Koga, F. A systematic review of serum gamma-glutamyltransferase as a prognostic biomarker in patients with genitourinary cancer. *Antioxid. (Basel)*. **10**, 549. 10.3390/antiox10040549 (2021). 10.3390/antiox10040549 PMC8066142 33916150

[7] Kocianova, E., Piatrikova, V. & Golias, T. Revisiting the Warburg effect with focus on lactate. *Cancers* **14**, 6028. 10.3390/cancers14246028 (2022). 36551514 10.3390/cancers14246028 PMC9776395

[8] Miholjcic, T. B. S. et al. Rationale for LDH-targeted cancer immunotherapy. *Eur. J. Cancer*. **181**, 166–178. 10.1016/j.ejca.2022.11.032 (2023). 36657325 10.1016/j.ejca.2022.11.032

[9] Gray, L. R., Tompkins, S. C. & Taylor, E. B. Regulation of pyruvate metabolism and human disease. *Cell. Mol. Life Sci.* **71**, 2577–2604. 10.1007/s00018-013-1539-2 (2014). 24363178 10.1007/s00018-013-1539-2 PMC4059968

[10] Helenius, I. T., Madala, H. R. & Yeh, J. J. An Asp to strike out cancer? Therapeutic possibilities arising from aspartate’s emerging roles in cell proliferation and survival. *Biomolecules* **11**, 1666. 10.3390/biom11111666 (2021). 34827664 10.3390/biom11111666 PMC8615858

[11] Holecek, M. Aspartic acid in health and disease. *Nutrients* **15**, 4023. 10.3390/nu15184023 (2023). 37764806 10.3390/nu15184023 PMC10536334

[12] Buerk, B. T. et al. Prognostic potential of standard laboratory parameters in patients with metastatic renal cell cancer receiving first-line immunotherapy. *Sci. Rep.* **14**, 25365. 10.1038/s41598-024-76928-3 (2024). 39455722 10.1038/s41598-024-76928-3 PMC11511985

[13] Shirotake, S. et al. Serum lactate dehydrogenase before nivolumab treatment could be a therapeutic prognostic biomarker for patients with metastatic clear cell renal cell carcinoma. *Anticancer Res.* **39**, 4371–4377. 10.21873/anticanres.13606 (2019). 31366532 10.21873/anticanres.13606

[14] Powles, T. et al. Nivolumab plus cabozantinib versus sunitinib for first-line treatment of advanced renal cell carcinoma: extended follow-up from the phase III randomised CheckMate 9ER trial. *ESMO Open.* **9**, 102994. 10.1016/j.esmoop.2024.102994 (2024). 38642472 10.1016/j.esmoop.2024.102994 PMC11046044

[15] Choueiri, T. K. et al. Lenvatinib plus pembrolizumab versus sunitinib as first-line treatment of patients with advanced renal cell carcinoma (CLEAR): extended follow-up from the phase 3, randomised, open-label study. *Lancet Oncol.* **24**, 228–238. 10.1016/S1470-2045(23)00049-9 (2023). 36858721 10.1016/S1470-2045(23)00049-9

[16] Choueiri, T. K. et al. Updated efficacy results from the JAVELIN Renal 101 trial: first-line avelumab plus axitinib versus sunitinib in patients with advanced renal cell carcinoma. *Ann. Oncol.* **31**, 1030–1039. 10.1016/j.annonc.2020.04.010 (2020). 32339648 10.1016/j.annonc.2020.04.010 PMC8436592

[17] Plimack, E. R. et al. Pembrolizumab plus axitinib versus sunitinib as first-line treatment of advanced renal cell carcinoma: 43-month follow-up of the phase 3 KEYNOTE-426 study. *Eur. Urol.* **84**, 449–454. 10.1016/j.eururo.2023.06.006 (2023). 37500340 10.1016/j.eururo.2023.06.006

[18] Shapiro, D. D. et al. Understanding the tumor immune microenvironment in renal cell carcinoma. *Cancers* **15**10.3390/cancers15092500 (2023). 10.3390/cancers15092500 PMC10177515 37173966

[19] Annels, N. E. et al. The dysfunctional immune response in renal cell carcinoma correlates with changes in the metabolic landscape of ccRCC during disease progression. *Cancer Immunol. Immunother*. **72**, 4221–4234. 10.1007/s00262-023-03558-5 (2023). 37940720 10.1007/s00262-023-03558-5 PMC10700462

[20] Santoni, M. et al. Global real-world outcomes of patients receiving immuno-oncology combinations for advanced renal cell carcinoma: The ARON-1 study. *Target. Oncol.* **18**, 559–570. 10.1007/s11523-023-00978-2 (2023). 37369815 10.1007/s11523-023-00978-2

[21] Shah, N. J. et al. Real-world treatment patterns and clinical outcomes for metastatic renal cell carcinoma in the current treatment era. *Eur. Urol. Open. Sci.* **49**, 110–118. 10.1016/j.euros.2022.12.015 (2023). 36874600 10.1016/j.euros.2022.12.015 PMC9974999

[22] Stuhler, V., Herrmann, L., Rausch, S., Stenzl, A. & Bedke, J. Real world data on IO-based therapy for metastatic renal cell carcinoma. *J. Cancer Res. Clin. Oncol.* **149**, 3249–3258. 10.1007/s00432-022-04173-0 (2023). 35907009 10.1007/s00432-022-04173-0 PMC10314860

[23] Kim, S. H. et al. Liver metastasis and Heng risk are prognostic factors in patients with non-nephrectomized synchronous metastatic renal cell carcinoma treated with systemic therapy. *PloS one*. **14**, e0211105. 10.1371/journal.pone.0211105 (2019). 30785902 10.1371/journal.pone.0211105 PMC6382149

[24] Tian, B. W. et al. Effect of liver metastasis on the efficacy of immune checkpoint inhibitors in cancer patients: a systemic review and meta-analysis. *Clin. Exp. Metastasis*. **40**, 255–287. 10.1007/s10585-023-10217-7 (2023). 37308706 10.1007/s10585-023-10217-7

[25] Ishiyama, Y. et al. Predictive role of gamma-glutamyltransferase in patients receiving nivolumab therapy for metastatic renal cell carcinoma. *Int. J. Clin. Oncol.* **26**, 552–561. 10.1007/s10147-020-01819-2 (2021). 33135126 10.1007/s10147-020-01819-2

[26] Yamamoto, Y. et al. Prognostic value of risk stratification using blood parameters for nivolumab in Japanese patients with metastatic renal-cell carcinoma. *Jpn J. Clin. Oncol.* **50**, 214–220. 10.1093/jjco/hyz168 (2020). 31755525 10.1093/jjco/hyz168

[27] Winter, J. et al. Prognostic role of gamma-glutamyl transferase in metastatic melanoma patients treated with immune checkpoint inhibitors. *Cancer Immunol. Immunother*. **70**, 1089–1099. 10.1007/s00262-020-02768-5 (2021). 33113003 10.1007/s00262-020-02768-5 PMC10991606

[28] Xu, L. et al. Alkaline phosphatase combined with gamma-glutamyl transferase is an independent predictor of prognosis of hepatocellular carcinoma patients receiving programmed death-1 inhibitors. *Front. Immunol.* **14**, 1115706. 10.3389/fimmu.2023.1115706 (2023). 36761721 10.3389/fimmu.2023.1115706 PMC9905229

[29] Zheng, Z. et al. A novel liver-function-indicators-based prognosis signature for patients with hepatocellular carcinoma treated with anti-programmed cell death-1 therapy. *Cancer Immunol. Immunother*. **73**, 158. 10.1007/s00262-024-03713-6 (2024). 38834790 10.1007/s00262-024-03713-6 PMC11150358

[30] Peng, L. et al. Peripheral blood markers predictive of outcome and immune-related adverse events in advanced non-small cell lung cancer treated with PD-1 inhibitors. *Cancer Immunol. Immunother*. **69**, 1813–1822. 10.1007/s00262-020-02585-w (2020). 32350592 10.1007/s00262-020-02585-w PMC7413896

[31] Xie, Z. et al. Targeting GGT1 eliminates the tumor-promoting effect and enhanced immunosuppressive function of myeloid-derived suppressor cells caused by G-CSF. *Front. Pharmacol.* **13**, 873792. 10.3389/fphar.2022.873792 (2022). 35548341 10.3389/fphar.2022.873792 PMC9081766

[32] Del Campo-Pedrosa, R., Martin-Carnicero, A., Gonzalez-Marcos, A. & Martinez, A. New model to predict survival in advanced pancreatic ductal adenocarcinoma patients by measuring GGT and LDH levels and monocyte count. *Front. Oncol.* **14**, 1411096. 10.3389/fonc.2024.1411096 (2024). 39435278 10.3389/fonc.2024.1411096 PMC11491290

[33] Liu, J. et al. Systematic oxidative stress indices predicts prognosis in patients with urothelial carcinoma of the upper urinary tract after radical nephroureterectomy. *Eur. J. Med. Res.* **28**, 469. 10.1186/s40001-023-01295-0 (2023). 37898799 10.1186/s40001-023-01295-0 PMC10612206

