# Supplementary material for: Combined elevation of pre-treatment γ-glutamyltransferase and lactate dehydrogenase as independent prognosticator for metastatic renal cell carcinoma undergoing immune-based therapy
Source: Sci Rep. 2026 Apr 18;16:12742. doi: 10.1038/s41598-026-48270-3 (PMC13091787; doi:10.1038/s41598-026-48270-3)
Supplement: Supplementary file 1 — Supplementary Material 1 [file 41598_2026_48270_MOESM1_ESM.pdf]

Scientific Reports (submitted in 2025) - Uhlig et al.

Combined elevation of pre-treatment γ-glutamyltransferase and lactate dehydrogenase as independent prognosticator for metastatic renal cell carcinoma undergoing immune-based therapy

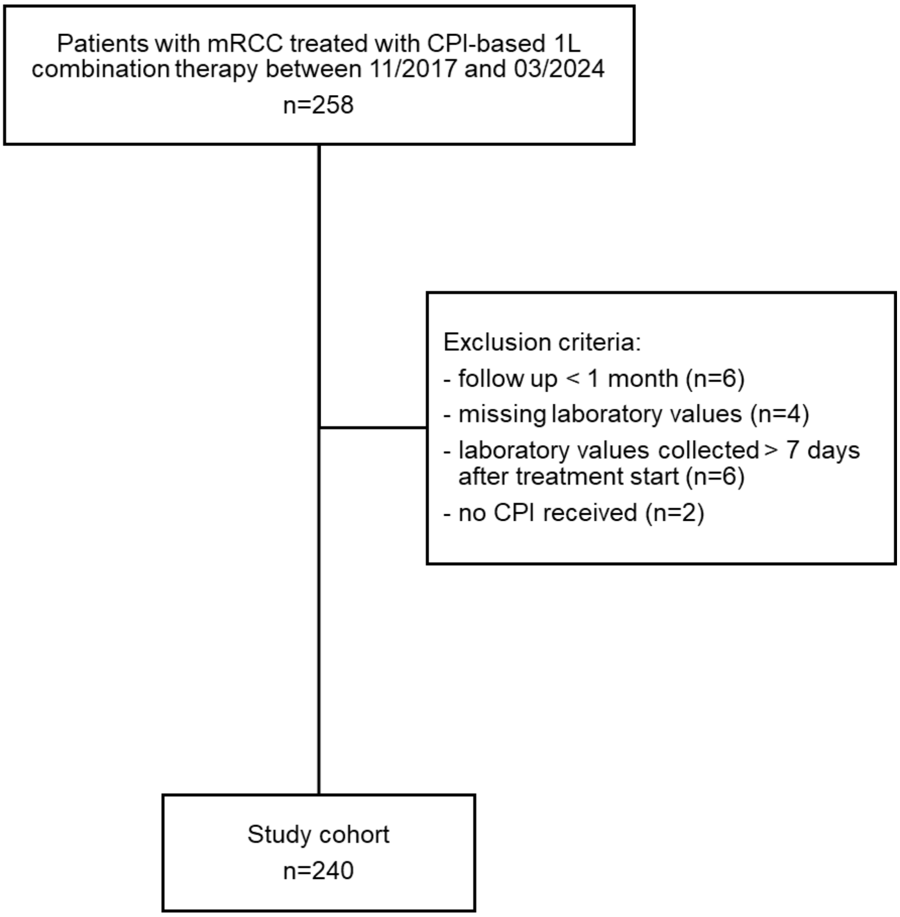

Fig. S1. Flow diagram for the composition of the study cohort.

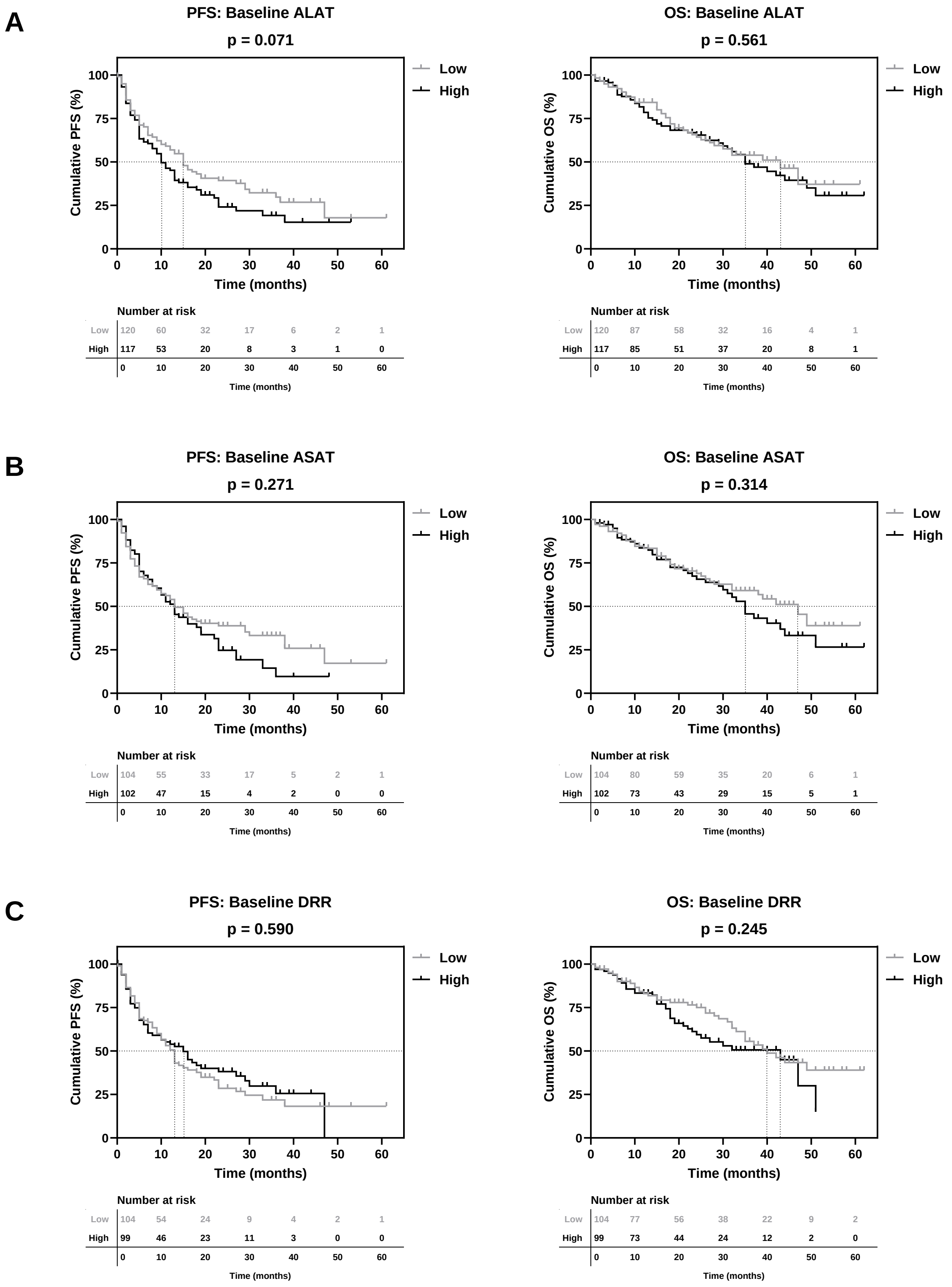

Fig. S2. Association of baseline (**A**) ALAT, (**B**) ASAT and (**C**) DRR with PFS and OS of patients with mRCC undergoing CPI-based 1L therapy. Vertical dashed lines indicate the respective median survival times of each category. P values were calculated using the log-rank test.

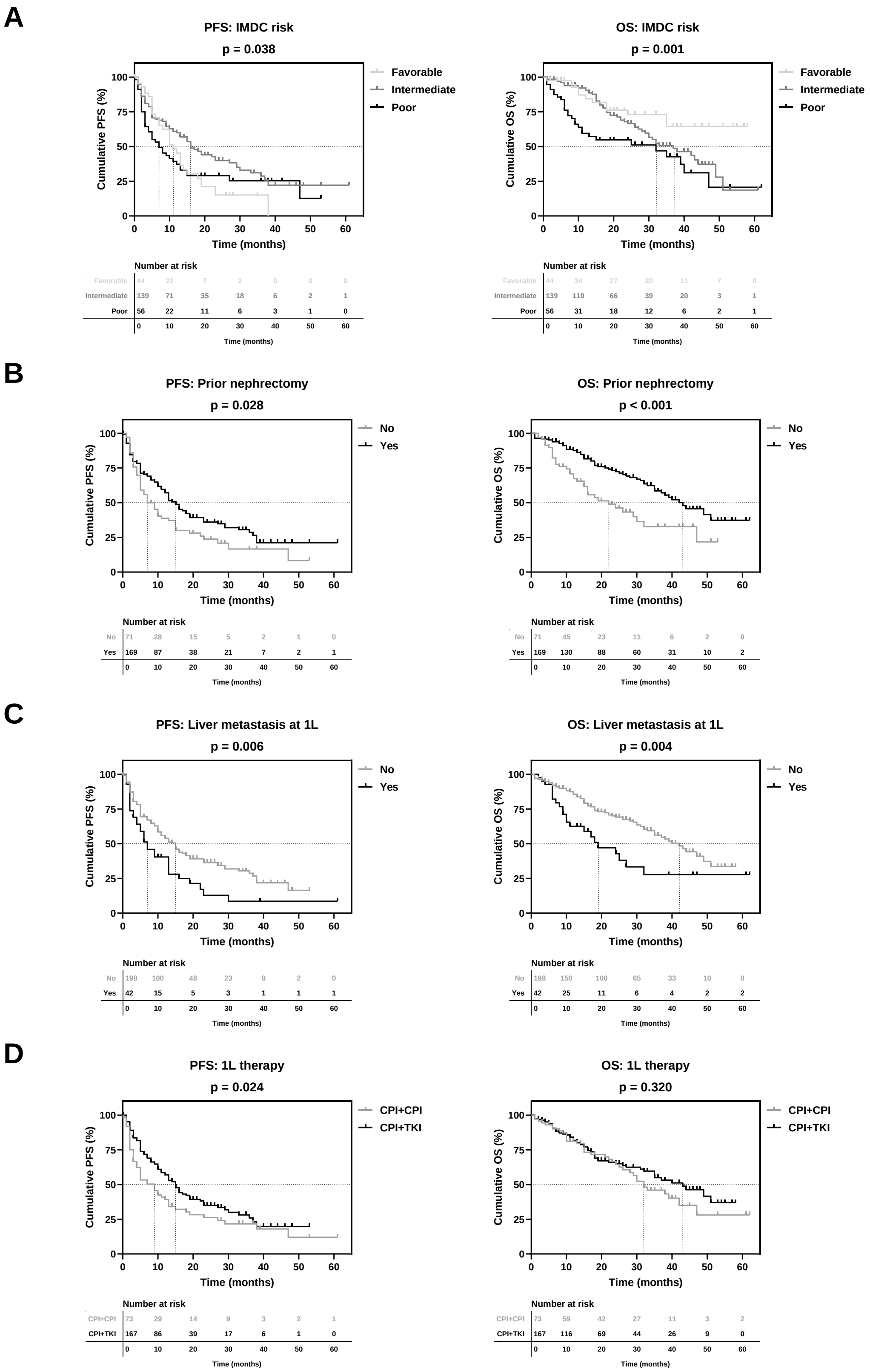

Fig. S3. Association of (**A**) IMDC risk, (**B**) nephrectomy status, (**C**) presence of liver metastasis and (**D**) type of combination therapy with PFS and OS of patients with mRCC undergoing CPI-based 1L therapy. Vertical dashed lines indicate the respective median survival time of each category. P values were calculated using the log-rank test.

A

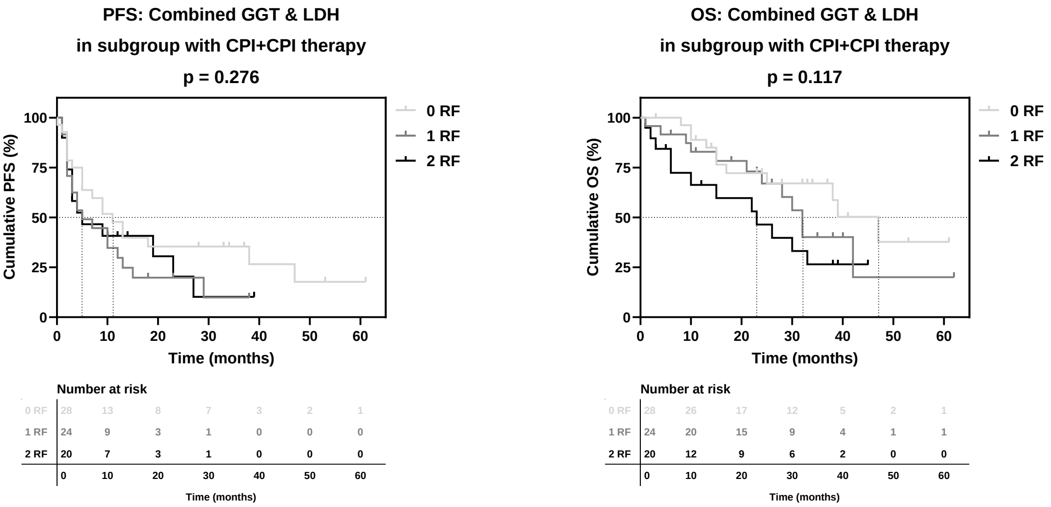

B

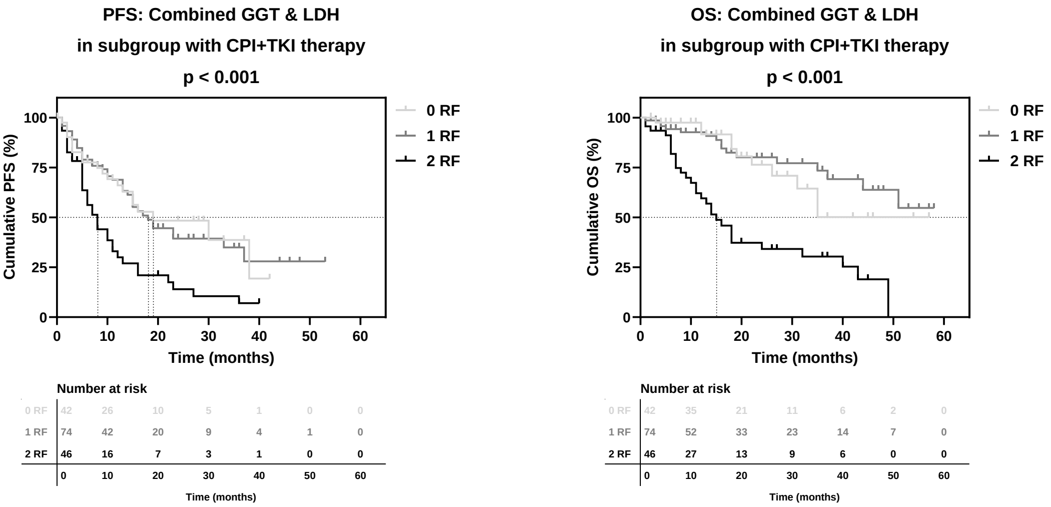

Fig. S4. Association of the combined baseline GGT/LDH risk groups with PFS and OS of patients with mRCC undergoing CPI-based 1L therapy in the treatment subgroups of (**A**) CPI+CPI and (**B**) CPI+TKI. A risk factor (RF) is defined as either high GGT or LDH. Vertical dashed lines indicate the respective median survival time of each category. P values were calculated using the log-rank test.

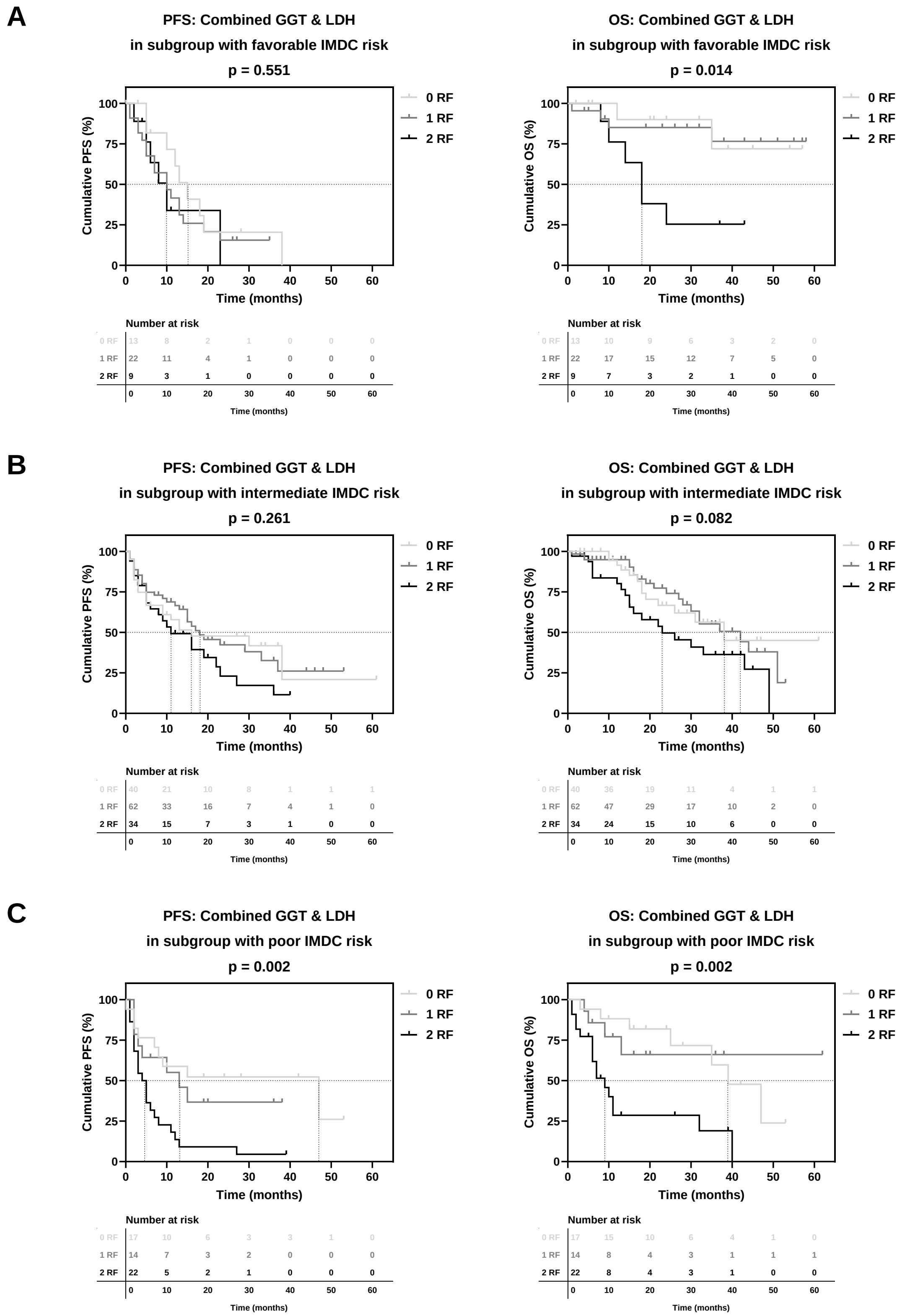

Fig. S5. Association of the combined baseline GGT/LDH risk groups with PFS and OS of patients with mRCC undergoing CPI-based 1L therapy in the subgroups of (**A**) favorable, (**B**) intermediate and (**C**) poor IMDC risk. A risk factor (RF) is defined as either high GGT or LDH. Vertical dashed lines indicate the respective median survival time of each category. P values were calculated using the log-rank test.

Table S1. Pearson correlation coefficients (r) indicating the relationships between two laboratory parameters at baseline. Significant p values (< 0.05) are displayed in bold.

	ALAT	ASAT	GGT
**ASAT**	r = 0.691<br>**p < 0.001**<br>n = 203	-	
**GGT**	r = 0.428<br>**p < 0.001**<br>n = 236	r = 0.425<br>**p < 0.001**<br>n = 205	-
**LDH**	r = 0.284<br>**p < 0.001**<br>n = 231	r = 0.473<br>**p < 0.001**<br>n = 202	r = 0.270<br>**p < 0.001**<br>n = 234

Table S2. Median baseline levels of ALAT, ASAT, DRR, GGT and LDH depending on the presence of liver metastasis. [a]Comparison between groups by the Mann-Whitney-U test.

Parameter	Category	No liver metastasis	Liver metastasis	P value [a]
**ALAT (U/l)**	n	42	195	0.153
	Median (range)	19.0 (5.0-115.8)	21.5 (5.0-113.0)	
**ASAT (U/l)**	n	37	169	0.224
	Median (range)	22.0 (8.4-145.2)	23.4 (11.4-163.2)	
**DRR**	n	37	166	0.598
	Median (range)	1.18 (0.27-4.40)	1.18 (0.62-3.80)	
**GGT (U/l)**	n	42	197	0.121
	Median (range)	34.0 (10.0-637.0)	36.5 (12.0-682.0)	
**LDH (U/l)**	n	42	192	0.160
	Median (range)	218 (44-848)	235 (131-989)	

Table S3. Number of patients and events as well as median survival times, 1- and 2-year survival rates for PFS and OS depending on the respective baseline categories of ALAT, ASAT, DDR, GGT and LDH as well as on the combined baseline GGT/LDH risk groups. A risk factor (RF) is defined as either high GGT or LDH. mo.: months, RF: risk factor.

Parameter	Category	PFS				OS			
		n (events)	1-year PFS	2-year PFS	Median PFS	n (events)	1-year OS	2-year OS	Median OS
**Baseline ALAT**	Low	120 (69)	59%	39%	15 mo.	120 (43)	84%	65%	43 mo.
	High	117 (78)	46%	24%	10 mo.	117 (50)	81%	66%	35 mo.
**Baseline ASAT**	Low	104 (64)	56%	39%	13 mo.	104 (39)	84%	70%	47 mo.
	High	102 (62)	52%	25%	13 mo.	102 (41)	83%	67%	35 mo.
**Baseline DRR**	Low	104 (68)	53%	29%	13 mo.	104 (39)	84%	76%	40 mo.
	High	99 (56)	55%	38%	15 mo.	99 (39)	83%	61%	43 mo.
**Baseline GGT**	Low	120 (67)	56%	40%	13 mo.	120 (35)	92%	75%	47 mo.
	High	119 (81)	50%	25%	12 mo.	119 (59)	74%	56%	30 mo.
**Baseline LDH**	Low	120 (69)	64%	39%	15 mo.	120 (38)	92%	76%	44 mo.
	High	114 (76)	41%	23%	9 mo.	114 (54)	74%	55%	30 mo.
**Combined GGT & LDH**	0 RF	70 (39)	60%	42%	15 mo.	70 (22)	94%	75%	47 mo.
	1 RF	98 (56)	60%	34%	15 mo.	98 (28)	90%	78%	51 mo.
	2 RF	66 (50)	35%	16%	8 mo.	66 (42)	63%	40%	16 mo.

Table S4. C-indices for clinico-pathological parameters, baseline laboratory parameters and the combined baseline GGT/LDH risk groups as well as for multivariate models with regards to PFS and OS (based on Tables 3, 4 & 5). NA: not applicable; Multivariate model 1 for PFS: nephrectomy status, presence of liver metastasis, type of 1L therapy, baseline LDH; Multivariate model 2 for PFS: nephrectomy status, presence of liver metastasis, type of 1L therapy, combined GGT & LDH; Multivariate model 1 for OS: IMDC risk group, nephrectomy status, presence of liver metastasis, baseline GGT, baseline LDH; Multivariate model 2 for OS: IMDC risk group, nephrectomy status, presence of liver metastasis, combined GGT & LDH.

Parameter / Model	C-indices for PFS (95%CI)	C-indices for OS (95%CI)
**IMDC risk**	NA	0.610 (0.548-0.673)
**Prior nephrectomy**	0.542 (0.500-0.583)	0.596 (0.543-0.649)
**Liver metastasis at 1L**	0.545 (0.509-0.581)	0.566 (0.519-0.613)
**1L therapy**	0.564 (0.521-0.608)	NA
**Baseline GGT**	NA	0.596 (0.542-0.650)
**Baseline LDH**	0.557 (0.511-0.603)	0.610 (0.558-0.663)
**Combined GGT & LDH**	0.564 (0.514-0.614)	0.642 (0.579-0.705)
**Multivariate model 1 for PFS**	0.619 (0.571-0.666)	NA
**Multivariate model 2 for PFS**	0.633 (0.584-0.682)	NA
**Multivariate model 1 for OS**	NA	0.693 (0.633-0.753)
**Multivariate model 2 for OS**	NA	0.704 (0.643-0.766)
